# A *Dictyostelium* Secreted Factor Requires a PTEN-Like Phosphatase to Slow Proliferation and Induce Chemorepulsion

**DOI:** 10.1371/journal.pone.0059365

**Published:** 2013-03-12

**Authors:** Sarah E. Herlihy, Yitai Tang, Richard H. Gomer

**Affiliations:** 1 Department of Biology, Texas A&M University, College Station, Texas, United States of America; 2 Department of Pathology, Stanford University Medical School, Stanford, California, United States of America; Cardiff University, United Kingdom

## Abstract

In *Dictyostelium discoideum*, AprA and CfaD are secreted proteins that inhibit cell proliferation. We found that the proliferation of cells lacking CnrN, a phosphatase and tensin homolog (PTEN)-like phosphatase, is not inhibited by exogenous AprA and is increased by exogenous CfaD. The expression of CnrN in *cnrN¯* cells partially rescues these altered sensitivities, suggesting that CnrN is necessary for the ability of AprA and CfaD to inhibit proliferation. Cells lacking CnrN accumulate normal levels of AprA and CfaD. Like cells lacking AprA and CfaD, *cnrN¯* cells proliferate faster and reach a higher maximum cell density than wild type cells, tend to be multinucleate, accumulate normal levels of mass and protein per nucleus, and form less viable spores. When *cnrN¯* cells expressing myc-tagged CnrN are stimulated with a mixture of rAprA and rCfaD, levels of membrane-associated myc-CnrN increase. AprA also causes chemorepulsion of *Dictyostelium* cells, and CnrN is required for this process. Combined, these results suggest that CnrN functions in a signal transduction pathway downstream of AprA and CfaD mediating some, but not all, of the effects of AprA and CfaD.

## Introduction

Much remains to be understood about how tissue size is regulated. A possible way to regulate tissue growth is through secreted autocrine factors that slow the proliferation of cells in that tissue. A variety of observations suggest the presence of such factors (often referred to as chalones) in many different tissues, but little is known about these factors and their signal transduction pathways [1], [2], [3], [4], [5]. Two such secreted autocrine factors have been identified in the model organism *Dictyostelium discoideum*. These proteins, AprA and CfaD, inhibit the proliferation of *Dictyostelium* cells as a population becomes dense. Strains lacking AprA or CfaD proliferate rapidly [6], [7], [8]. Cell proliferation is slowed by adding recombinant AprA (rAprA) or recombinant CfaD (rCfaD) to cells, or by overexpressing these proteins [6], [9]. Both *aprA¯* and *cfaD¯* cells are multinucleate [6], [7]. Although AprA and CfaD affect proliferation, cells lacking these proteins show mass and protein accumulation on a per nucleus basis similar to that of wild type cells, indicating that AprA and CfaD do not affect the growth of cells [6], [7]. In addition to inhibiting proliferation, AprA also causes chemorepulsion of cells, suggesting that AprA helps to disperse a colony of cells [10]. When starved, *Dictyostelium* cells develop to form fruiting bodies containing spores that can be dispersed to areas with higher nutrient concentrations. The ability to form viable spores is therefore advantageous. Although AprA and CfaD slow proliferation and appear to be deleterious, these proteins help spore development and are thus advantageous for development [6], [7].

During development, *Dictyostelium* cells aggregate using relayed pulses of cAMP as a chemoattractant. The cells move up a gradient of extracellular cAMP by extending pseudopods in the direction of the cAMP source [11], [12], [13]. Phosphatidylinositol 3-kinase (PI3K), which phosphorylates the membrane lipid phosphatidylinositol 4,5-bisphosphate (PIP_2_) to phosphatidylinositol 3,4,5-trisphosphate (PIP_3_), translocates from the cytosol to the membrane at the leading edge of the cell in response to cAMP and mediates actin polymerization and pseudopod formation [14], [15], [16], [17]. PTEN negatively regulates the effect of PI3K by dephosphorylating PIP_3_ to PIP_2_ [13], [14], [18]. When PTEN is localized to the membrane of cells, it inhibits the formation of pseudopods [13], [14], [15]. When PI3K translocates to the leading edge and PTEN localizes to the back edge of the cell, pseudopod formation is inhibited at the back of the cell, enabling movement toward cAMP [11], [15], [19].

CnrN is a PTEN-like protein in *Dictyostelium* that has PTEN-like phosphatase activity [20], [21]. In the absence of CnrN, levels of PIP_3_ are higher than in wild-type cells [20]. Akt, a downstream target in PI3K pathways, usually requires translocation and phosphorylation from the cytosol to the membrane for its activation [22], [23], [24]. During development, Akt translocation and phosphorylation is increased in the absence of CnrN [20]. The increases in Akt translocation, Akt phosphorylation, and levels of PIP_3_ in *cnrN¯* cells suggests that CnrN acts as a negative regulator of PIP_3_ and Akt, which are both components of PI3K pathways [20], [24]. Like PTEN, CnrN plays a role in *Dictyostelium* development. By antagonizing the PI3K pathway, CnrN negatively regulates the production of cAMP and stream breakup during aggregation [20]. Compared to wild type cells, *cnrN¯* cells have smaller aggregation territories and fruiting bodies, increased cell motility, number of aggregation territories, cAMP levels, Akt translocation, and actin polymerization, move faster, move further toward cAMP, are insensitive to counting factor, and have shorter streams [20]. All of these phenotypes are rescued by the expression of a myc-tagged CnrN in the *cnrN¯* cells (*cnrN¯*/*CnrN^OE^*) [20].

In mammalian cells, PI3K signaling leads to cell proliferation [18], [25], [26]. PIP_3_ binds to downstream effectors such as PDK1 and Akt, which play a role in cell proliferation, growth, and survival [18], [25], [27], [28], [29]. As PTEN effectively counteracts PI3K, it negatively regulates proliferation in mammalian systems and functions as a tumor suppressor [18], [30], [31], [32], [33]. Here we report that CnrN is necessary for the inhibition of proliferation by AprA and CfaD as well as AprA-induced chemorepulsion, indicating that CnrN acts as a negative regulator of proliferation in a chalone signal transduction pathway and mediates AprA-induced chemorepulsion.

## Materials and Methods

Ax2 wild-type and *cnrN¯* clone DBS0302655 [20] were grown in HL5 media (Formedium Ltd, Norwich, England) as previously described [34]. *cnrN¯/CnrN^OE^* clone DBS0302656 and Ax2*/CnrN^OE^* clone YT05A cells were cultured in HL5 containing 15 µg/ml geneticin [20]. Recombinant AprA and CfaD were made following Bakthavatsalam et al. [6]. Levels of extracellular AprA and CfaD were compared by starting cultures of cells in axenic shaking culture at 1.25×10^6^ cells/ml and collecting cells by centrifugation at 3000×g for 4 minutes when cultures were at 2×10^6^ cells/ml. A sample of the conditioned medium supernatant was mixed with an equal volume of 2X loading buffer and heated to 95°C for 5 minutes. 10 μl of these samples were run on 4–20% polyacrylamide gels, and Western blots were stained for AprA as previously described [7] or for CfaD as previously described [6]. DAPI staining of nuclei was done as described [7]. Proliferation inhibition, spore viability, mass and protein determination, doubling time calculations, and statistics were done as previously described [3] with the exception that for spore viability assays, 10^7^ cells were washed twice in 8 ml PDF (20 mM KCl, 9.2 mM K_2_HPO_4_, 13.2 mM KH_2_PO_4_, 1 mM CaCl_2_, 2.5 mM MgSO_4_, pH 6.4) prior to resuspension. In addition, proliferation inhibition was measured using a combination of rAprA and rCfaD, both at 300 ng/ml. Fluorescence microscopy was conducted following [35], [36]. Briefly, vegetative cells were placed in 8-well chambered glass slides (Nalge Nunc 177402) at a density of 5×10^4^ cells/well, fixed with 3.7% formaldehyde for 10 minutes, and permeabilized with 0.5% TritonX-100 in PBS (140 mM NaCl, 2.7 mM KCl, 10 mM Na_2_HPO_4_, 1.8 mM KH_2_PO_4_, pH 7.4) for 5 minutes. Cells were stained with a 30-minute incubation with anti-Myc antibodies (Bethyl Laboratories) and a subsequent 30-minute incubation with Alexa Fluor 488 conjugated anti-rabbit IgG (Invitrogen) in PBS containing 0.05% NP-40 and 0.5% BSA at room temperature. Images were taken using an Axioplan Fluorescence Microscope (Carl Zeiss). Binding of AprA, CfaD, a mixture of both, or an equal volume of buffer to myc-tagged *cnrN¯/CnrN^OE^* cells was done as previously described [9], [37]. Membranes were collected as previously described [9], [37], and Western blots of membrane samples were stained with anti-Myc antibodies. Membranes were re-probed with anti-AprA and anti-CfaD antibodies (Bethyl Laboratories). Chemorepulsion assays were done as previously described [10].

## Results

### *cnrN¯* cells show aberrant proliferation inhibition by AprA and CfaD

**Figure 1:**
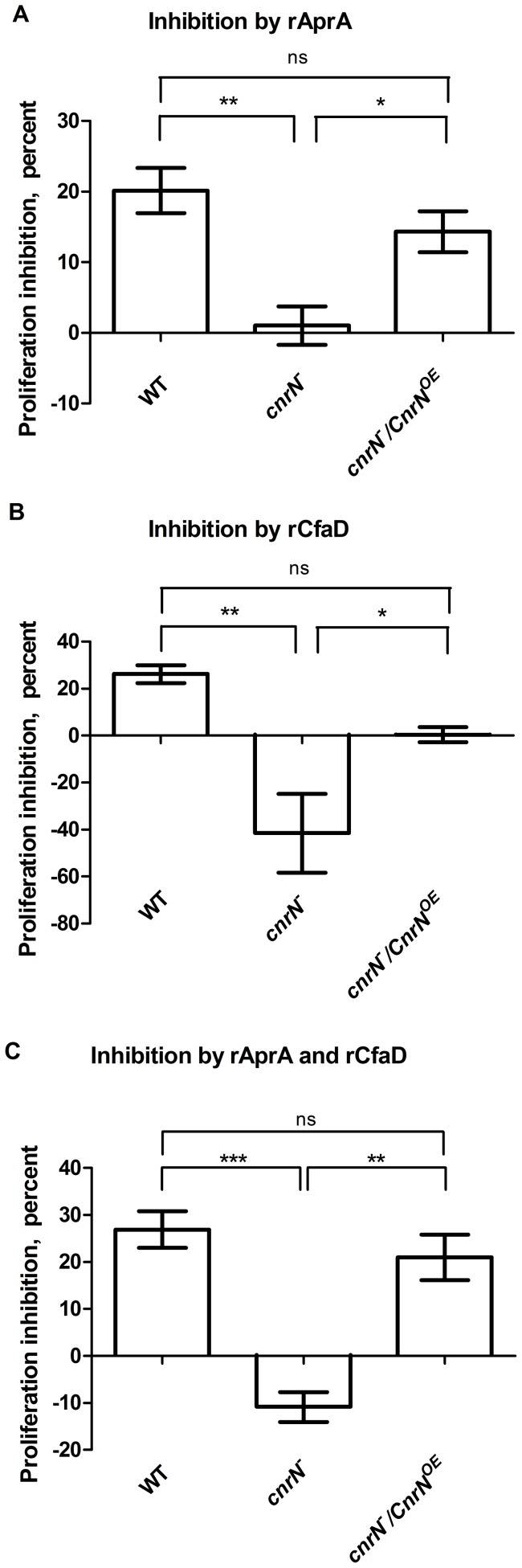
*cnrN¯* cells are insensitive to rAprA and have altered sensitivity to rCfaD. Cell densities were measured after a 16-hour incubation with 300 ng/ml of rAprA or rCfaD, a combination of 300 ng/ml rAprA and 300 ng/ml rCfaD, or an equivalent volume of buffer. The percent of proliferation inhibition by (**A**) rAprA, (**B**) rCfaD, or (**C**) rAprA and rCfaD compared to the proliferation of the buffer control is shown. Values are mean ± SEM from at least three independent experiments. * indicates that the difference is statistically significant at p<0.05, ** indicates p<0.01, *** indicates p<0.001, and ns indicates not significant (one-way ANOVA, Tukey's test). Compared to the addition of an equal volume of buffer, the addition of rCfaD or the combination of rAprA and rCfaD to *cnrN¯* cells significantly increased proliferation (t-test, p<0.05).

The proliferation of wild-type cells is inhibited by AprA or CfaD [6], [7], [9]. If AprA and/or CfaD transduce signaling through CnrN, we would expect *cnrN¯* cells to be insensitive to AprA and/or CfaD. We incubated proliferating cells with rAprA or rCfaD and determined the decrease in cell density compared to a buffer control after a 16-hour incubation. As previously observed, wild-type cells had an approximately 20 percent decrease in proliferation in response to rAprA or rCfaD (Figure 1) [2], [3]. *cnrN¯* cells were essentially insensitive to rAprA, and this phenotype was rescued by expressing CnrN in the mutant background (Figure 1A). Compared to the addition of buffer, the addition of rCfaD to *cnrN¯* cells significantly increased their proliferation (Figure 1B). Expressing CnrN in the *cnrN¯* background blocked the ability of rCfaD to increase proliferation, but did not restore the ability of rCfaD to inhibit proliferation (Figure 1B). A combination of rAprA and rCfaD inhibited wild type proliferation similarly to either recombinant protein alone (Figure 1C). The response of *cnrN¯* cells to the combination of rAprA and rCfaD mimicked their response to rCfaD alone (Figure 1C). The difference in the increase of proliferation of *cnrN¯* cells between the addition of rCfaD alone and the combination of rCfaD and rAprA is statistically significant (t-test, p<0.05). The proliferation of *cnrN¯/CnrN^OE^* cells was inhibited by the combination of rAprA and rCfaD (Figure 1C). Together, these results suggest that CnrN is necessary for AprA and CfaD to inhibit proliferation.

### *cnrN¯* cells have fast proliferation

**Figure 2:**
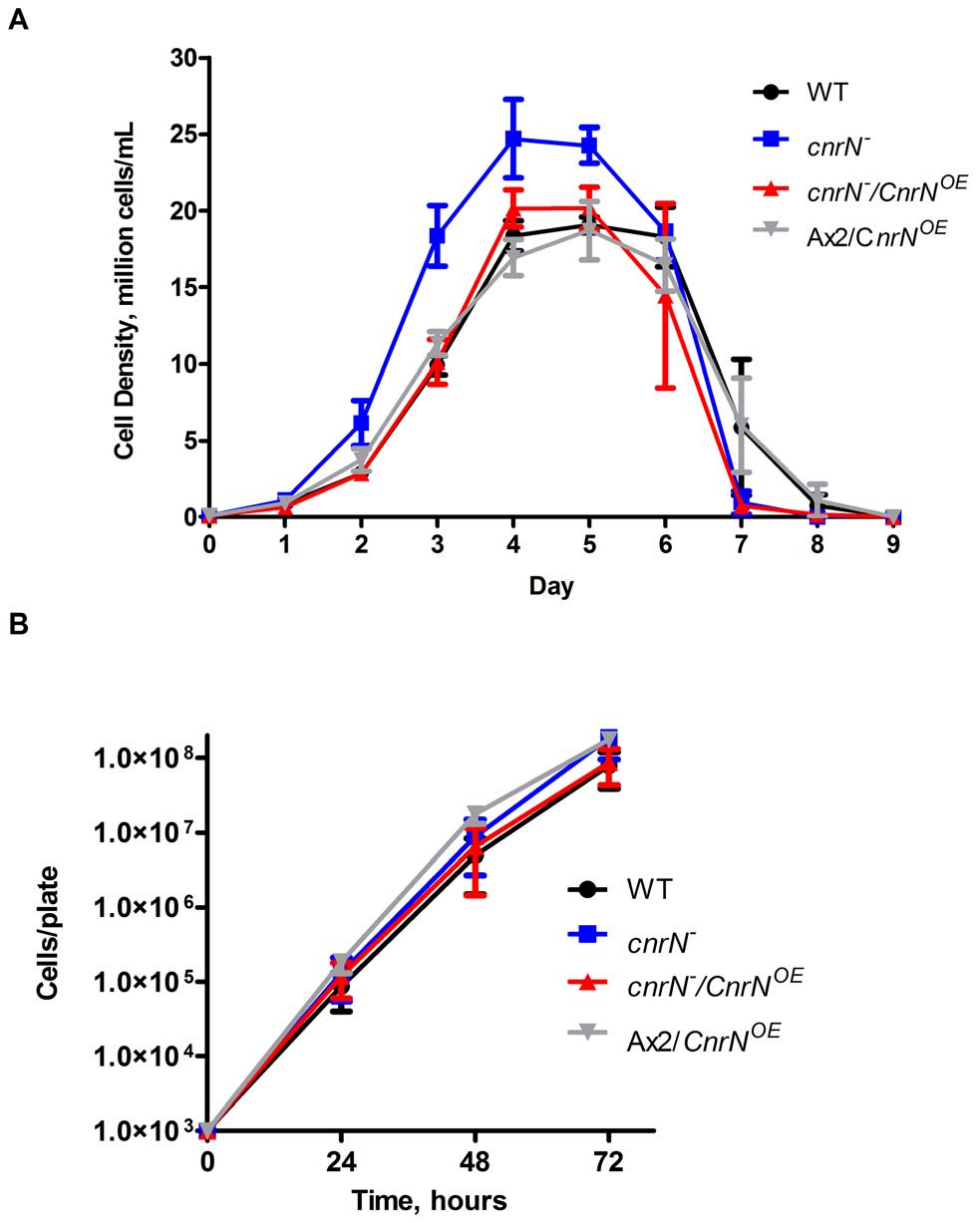
CnrN affects cell proliferation. (**A**) Log phase cells were inoculated into HL5 media at 1×10^5^ cells/ml and cell densities were measured daily. Values are mean ± SEM, n = 3 or more for all conditions. WT indicates wild type. (**B**) 1000 cells were plated on SM/5 plates with *K. aerogenes* bacteria and the total number of cells was determined daily. By 72 hours, cells had begun to overgrow the bacteria. Values are mean ± SEM, n = 3 for all conditions. The absence of error bars indicates that the error was smaller than the plot symbol. For cells grown on bacteria, there were no statistically significant differences in cell density at any time between the four strains (1-way ANOVA, Tukey's test).

Cells lacking AprA or CfaD proliferate faster and to a higher stationary density then wild-type cells [6], [7]. Like *aprA¯* and *cfaD¯* cells, *cnrN¯* cells proliferate faster, have a faster doubling time, and reach a higher maximum cell density than wild type cells (Figure 2A and Table 1). Occasionally, for unknown reasons, we observed this and other clones of *cnrN¯* cells proliferating slower than wild type cells. There are many reasons a mutant might proliferate slower, such as defects in a metabolic pathway, but it is unusual to find clones that proliferate faster than wild type. Therefore, we believe the fast proliferation phenotype is the true phenotype of *cnrN¯* cells. *cnrN¯/CnrN^OE^* and Ax2*/CnrN^OE^* cells showed no significant difference in doubling time or maximal cell density compared to wild type (Figure 2A and Table 1). Although *cnrN¯/CnrN^OE^* and Ax2*/CnrN^OE^* cells appeared to proliferate slower and to a lower cell density than *cnrN¯* cells, only the differences between the maximum cell densities were statistically significant. Unlike the proliferation in liquid culture, wild type, *cnrN¯*, *cnrN¯/CnrN^OE^*, and Ax2*/CnrN^OE^* cells showed similar proliferation rates on bacteria (Figure 2B). Together, the data suggest that in shaking liquid culture, CnrN decreases the proliferation of cells.

**Table 1:** The effect of CnrN on doubling time and stationary density of cells.

Cell Type	Doubling time, hours	Maximum observed cell density, 10^6^ cells/ml
Wild-type	13.7±1.1	20.2±0.6
*cnrN¯*	10.9±0.2*	27.9±1.4***
*cnrN¯/CnrN^OE^*	12.2±0.8	21.6±0.7
Ax2*/CnrN^OE^*	12.8±0.5	19.8±1.5

Doubling times and stationary densities were measured for the data shown in Figure 2A. Values are mean ± SEM from at least 3 independent experiments. * indicates values are significantly different from wild-type with p<0.05 (one-way ANOVA, Dunnett's test). *** indicates values are significantly different compared to wild type with p<0.001 (one-way ANOVA, Tukey's test). The difference in maximum cell density is statistically significant between *cnrN¯* and *cnrN¯/CnrN^OE^* with p<0.01 and between *cnrN¯* and Ax2*/CnrN^OE^* with p<0.001 (one-way ANOVA, Tukey's test).

### *cnrN¯* cells secrete AprA and CfaD

**Figure 3:**
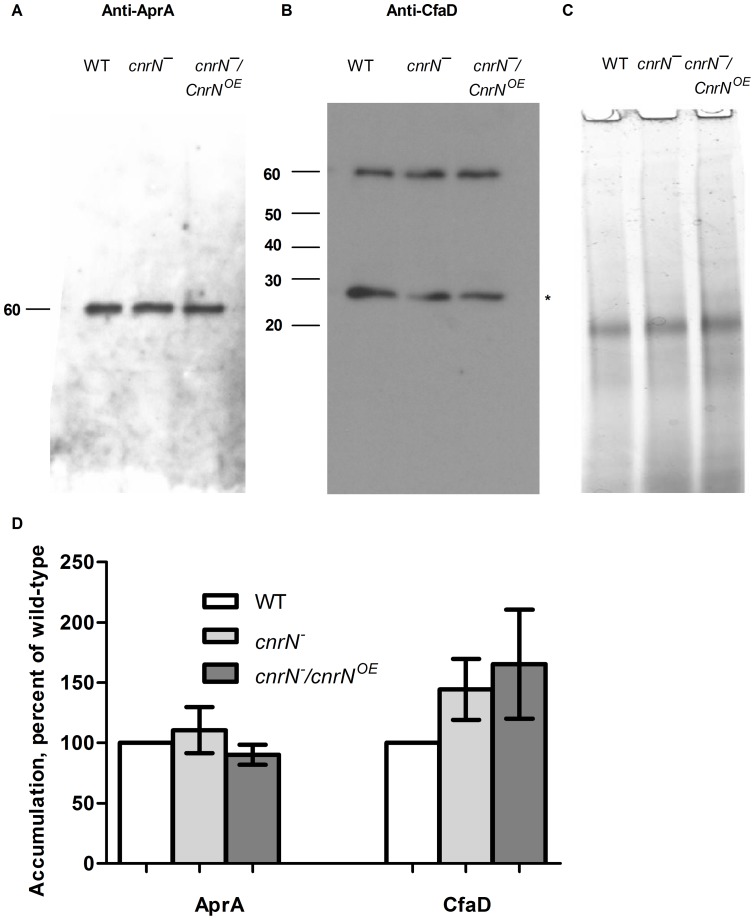
Cells lacking and overexpressing CnrN secrete normal levels of extracellular AprA and CfaD. Conditioned media from the indicated cell lines was assayed by Western blot with anti-AprA (**A**) or anti-CfaD (**B**) antibodies. Data is representative of four separate experiments. Molecular weights in kDa are shown at the left of the blots. Asterisk indicates a 27-kDa breakdown product of CfaD whose amount varied somewhat between cell lines and between experiments [5]. (**C**) Conditioned medium samples were run on a 4-20% PAGE gel and silver stained as a loading control. (**D**) Quantification of AprA and CfaD accumulation. Autoradiogram densities were normalized to the wild-type value, and are mean ± SEM. n = 4.

One explanation for the fast proliferation phenotype of *cnrN¯* cells could be a decrease in extracellular accumulation of AprA or CfaD. To test this hypothesis, we examined the extracellular levels of AprA and CfaD in *cnrN¯* and *cnrN¯/CnrN^OE^* cells. Both AprA and CfaD accumulate in the medium to levels that are comparable to wild-type levels (Figure 3). Although CfaD accumulation appears to be increased in *cnrN¯* and *cnrN¯/CnrN^OE^* cells, this increase is not significant compared to wild type accumulation (Figure 3D). This suggests that the fast proliferation phenotype of *cnrN¯* cells is not due to a decrease in extracellular levels of AprA or CfaD.

### *cnrN¯* cells are multinucleate

Both *aprA¯* and *cfaD¯* cells tend to be multinucleate [6], [7]. To determine if *cnrN¯* cells exhibit the same phenotype, the number of nuclei per cell was measured. Compared to wild-type cells, *cnrN¯* cells have significantly fewer cells with one nucleus per cell and significantly more cells with two nuclei or three or more nuclei per cell (Table 2). Expression of CnrN in the *cnrN¯* background partially rescued this phenotype. Expression of CnrN in a wild type background caused no significant change in the number of nuclei per cell compared to wild type. These results suggest that like AprA and CfaD, CnrN may be involved in cytokinesis.

**Table 2:** The effect of CnrN on the number of nuclei per cell.

	Percent of cells with n nuclei		
Cell Type	1	2	3+
Wild-type	77.9±1.8	19.0±2.1	3.1±0.3
*cnrN¯*	49.1±4.4***	32.2±3.9*	19.3±2.2***
*cnrN¯/CnrN^OE^*	55.6±0.8***	30.8±2.1*	13.6±1.9**
Ax2*/CnrN^OE^*	74.8±1.5	23.3±1.0	1.8±0.5

Fluorescence microscopy was used to count the number of nuclei per cell (at least 200 cells for each condition) for log phase cells stained with DAPI. Values are mean ± SEM from at least three independent experiments. * indicates value is significantly different compared to wild-type value at p<0.05, ** indicates p<0.01, and *** indicates p<0.001 (one-way ANOVA, Tukey's test). The difference in the number of cells with 1 nucleus or 3 or more nuclei was significantly different between *cnrN¯* and Ax2*/CnrN^OE^* cells with p<0.001 and between *cnrN¯/CnrN^OE^* and Ax_2_ */CnrN^OE^* cells with p<0.01 (one-way ANOVA, Tukey's test).

### CfaD decreases the multinucleate phenotype of *cnrN¯* cells

The addition of rCfaD to *cnrN¯* cells increases their proliferation (Figure 1). One explanation for this increase in proliferation is that rCfaD increases the cytokinesis of *cnrN¯* cells, which are more multinucleate than wild type cells. To test this possibility, wild type and *cnrN¯* cells were incubated with and without rCfaD for 16 hours. After verifying that rCfaD decreased the proliferation of wild type cells and increased the proliferation of *cnrN¯* cells, cells were stained with DAPI, and the nuclei per cell were counted. rCfaD did not cause a significant change in the nuclear phenotype of wild type cells. Adding rCfaD to *cnrN¯* cells significantly increased the number of cells with a single nucleus and significantly decreased the number of cells with two nuclei and cells with three or more nuclei compared to *cnrN¯* cells with no rCfaD (Table 3). The addition of rCfaD to either wild type or *cnrN¯* cells significantly decreased the number of nuclei per 100 cells (Table 3). Together, these data indicate that rCfaD reduces the multinuclearity of *cnrN¯* cells, presumably by increasing cytokinesis, and this would account for the increase in proliferation during inhibition assays.

**Table 3:** The effect of rCfaD on the number of nuclei per cell.

		Percent of cells with n nuclei			Nuclei/100 cells
Cell Type	rCfaD	1	2	3+	
Wild-type	**−**	85.8±2.1	13.6±2.0	0.6±0.4	115±2
Wild-type	**+**	87.9±1.5	11.6±1.2	0.6±0.4	112±2*
*cnrN¯*	**−**	50.6±2.8	41.5±1.7	7.9±1.5	164±9
*cnrN¯*	**+**	67.1±5.3*	28.6±3.2*	4.3±2.1*	138±8***

Following a 16-hour incubation with 300 ng/ml of rCfaD or an equivalent volume of buffer, fluorescence microscopy was used to count the number of DAPI-stained nuclei per cell (at least 200 cells for each condition). Values are mean ± SEM from three independent experiments. * and *** indicate that values are statistically significant between – and + rCfaD for the genotype with p<0.05 and p<0.001, respectively (one-way ANOVA, Tukey's test or one-way paired t-test).

### CnrN does not regulate growth on a per nucleus basis

Cell proliferation is defined as an increase in cell number, and cell growth as the accumulation of mass and protein [2], [3], [6], [7]. Both *aprA¯* and *cfaD¯* cells tend to be more massive than wild type, but do not accumulate mass or protein at an increased rate on a per nucleus basis compared to control [6], [7]. This indicates that neither AprA nor CfaD play a role in the regulation of growth. Mass and protein content was analyzed in log phase cells to determine the effect of CnrN on growth. Mass, protein, and nuclei per 100 cells for wild type were similar to previous observations [2], [3], [6], [7]. There was no statistically significant difference in mass between wild type, *cnrN¯*, *cnrN¯/CnrN^OE^*, or Ax2*/CnrN^OE^* cells (Table 4). Protein content and nuclei per 100 cells were increased compared to wild type in both *cnrN¯* and *cnrN¯/CnrN^OE^* cells. Protein content and protein content on a per nucleus basis was decreased in Ax2*/CnrN^OE^* cells. On a per nucleus basis, *cnrN¯* cells contained significantly less mass and protein than wild-type. *cnrN¯/CnrN^OE^* cells had significantly more protein content per nucleus than *cnrN¯* cells, rescuing the mutant phenotype.

**Table 4:** The effect of CnrN on the mass and protein content of cells.

	Per 10^7^ cells			Per 10^7^ nuclei	
Cell type	Mass (mg)	Protein (mg)	Nuclei/100 cells	Mass (mg)	Protein (mg)
Wild-type	10.3±0.4	0.86±0.02	126±1	8.2±0.3	0.68±0.02
*cnrN¯*	9.0±0.4	0.97±0.01**	187±7***	4.8±0.2***	0.52±0.02**
*cnrN¯/CnrN^OE^*	11.2±1.0	1.30±0.04***	170±4***	6.6±0.6	0.76±0.03
Ax2*/CnrN^OE^*	9.4±1.3	0.70±0.02***	131±6	7.1±0.9	0.53±0.02**

Mass and protein content was measured, and the data from Table 2 were use to determine the number of nuclei per 100 cells. Values are mean ± SEM from at least three independent experiments. ** indicates value is significantly different compared to wild type at p<0.01 and *** indicates p<0.001 (one-way ANOVA, Tukey's test). The difference in protein per 10^7^ cells and protein per 10^7^ nuclei is significantly different with p<0.001 between *cnrN¯* and *cnrN¯/CnrN^OE^* and between *cnrN¯/CnrN^OE^* and Ax2*/CnrN^OE^*. The difference in protein per 10^7^ cells is significantly different between *cnrN¯* and Ax2*/CnrN^OE^* with p<0.001. Both *cnrN¯* and *cnrN¯/CnrN^OE^* nuclei per 100 cells are significantly different from Ax2*/CnrN^OE^* with p<0.001.

To estimate mass, protein, and nuclei accumulation per hour, we assumed that a doubling in cell number also results in a doubling of mass, protein, and nuclei. We then divided the mass, protein, and nuclei contents for each genotype by their respective calculated doubling times (Table 1). On a per cell per hour basis, *cnrN¯*, *cnrN¯/CnrN^OE^*, and Ax2*/CnrN^OE^* cells accumulated mass similarly to wild-type (Table 5). Both *cnrN¯* and *cnrN¯/CnrN^OE^* cells accumulated significantly more protein per cell per hour than either wild type or Ax2*/CnrN^OE^* cells. *cnrN¯* and *cnrN¯/CnrN^OE^* cells accumulated nuclei at a faster rate than wild-type. *cnrN¯/CnrN^OE^* cells accumulated nuclei at a significantly slower rate compared to *cnrN¯* cells, partially rescuing the fast nuclei accumulation of *cnrN¯* cells. Ax2*/CnrN^OE^* cells accumulated nuclei at a rate similar to wild type, and this rate was significantly slower than the *cnrN¯* and *cnrN¯/CnrN^OE^* nuclei accumulation rates (Table 5). On a per nucleus basis, there were no significant differences in the mass and protein accumulation per hour in wild type, *cnrN¯*, *cnrN¯/CnrN^OE^*, and Ax2*/CnrN^OE^* cells. These results suggest that like AprA and CfaD, CnrN does not affect growth on a per nucleus basis.

**Table 5:** The effect of CnrN on mass and protein accumulation of cells.

	Per 10^7^ cells/hour			Per 10^7^ nuclei/hour	
Cell type	Mass (mg)	Protein (µg)	Nuclei, ×10^−5^	Mass (mg)	Protein (µg)
Wild-type	0.75±0.07	62.6±5.4	9.2±0.77	0.60±0.05	50±4
*cnrN¯*	0.82±0.04	88.6±2.1*	17±0.47***	0.44±0.02	47±1
*cnrN¯/CnrN^OE^*	0.92±0.10	107±7.4***	14±0.94***	0.54±0.06	63±5
Ax2*/CnrN^OE^*	0.87±0.08	54.2±2.5	10±0.61	0.67±0.07	41±3

Mass, protein, and nuclei values from Table 4 were divided by the doubling time for each respective genotype from Table 1. Values are mean ± SEM from at least three independent experiments. * indicates value is significantly different compared to wild-type value at p<0.05 and *** is significant at p<0.001 (one-way ANOVA, Tukey's test). The difference in protein per cell per hour is significantly different between *cnrN¯* and Ax2*/CnrN^OE^* and between *cnrN¯/CnrN^OE^* and Ax2*/CnrN^OE^* with p<0.01 and p<0.001, respectively. The differences in nuclei per 10^7^ cells per hour are significantly different between *cnrN¯* and *cnrN¯/CnrN^OE^*, *cnrN¯* and Ax2*/CnrN^OE^*, and *cnrN¯/CnrN^OE^* and Ax2*/CnrN^OE^* with p<0.001. The difference in protein per 10^7^ nuclei per hour is significantly different between *cnrN¯/CnrN^OE^* and Ax2*/CnrN^OE^* with p<0.05.

### CnrN affects spore viability

Cells lacking AprA and CfaD have reduced spore viability [6], [7]. *cnrN¯* cells were examined for their ability to form detergent-resistant spores. An equivalent number of wild-type, *cnrN¯*, *cnrN¯/CnrN^OE^*, and Ax2*/CnrN^OE^* cells were developed, the spores were collected, and dilutions of detergent-treated spores were plated. The numbers of cells with a spore morphology and the numbers of detergent-resistant spores from *cnrN¯* cells was significantly less compared to those recovered from wild type (Table 6). Expression of CnrN in the *cnrN¯* background rescued the number of detergent-resistant spores and appeared to partially rescue the number of visible spores. The number of recovered spores and the number of viable spores from *cnrN¯/CnrN^OE^* or Ax2*/CnrN^OE^* cells were similar to wild type levels. These data indicate that like AprA and CfaD, CnrN affects the ability of cells to form viable spores.

**Table 6:** The effect of CnrN on spore viability.

Cell type	Number of spores after development as a percent of input cell number	Detergent-resistant spores as a percent of total spores
Wild-type	100±12	83.3±6.3
*cnrN¯*	41.3±8.0**	37.8±2.5***
*cnrN¯/CnrN^OE^*	59.9±2.6	66.5±15
Ax2*/CnrN^OE^*	109±11	107±16

Cells were developed on filter pads for 48 hours and the percent of visible spores was calculated as a percent of input cells. Detergent-treated spores were plated and analyzed for their ability to produce colonies. Values are mean ± SEM from at least three independent experiments. ** and *** indicates differences are significantly different compared to wild-type with p<0.01 and p<0.001, respectively (one-way ANOVA, Tukey's test). The difference in the number of visible spores between *cnrN¯* and Ax2*/CnrN^OE^* is significantly different with p<0.01. The difference in the number of detergent-resistant spores between *cnrN¯* and *cnrN¯/CnrN^OE^* is significantly different with p<0.05. The number of detergent-resistant spores in Ax2*/CnrN^OE^* is significantly different from both *cnrN¯* (p<0.001) and *cnrN¯/CnrN^OE^* (p<0.05).

### AprA and CfaD affect CnrN localization

**Figure 4:**
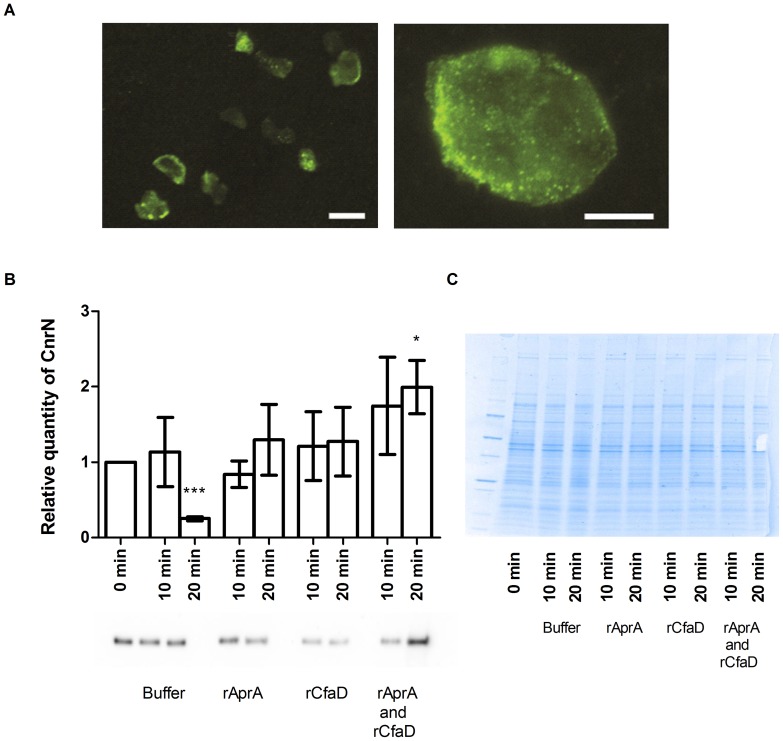
CnrN localization. (**A**) CnrN localization was determined by staining myc-tagged *cnrN¯/CnrN^OE^* cells for the myc-tag and fluorescent images were taken using a 20× (left) or 100x (right) lens. Size bars are 10 µm (left) and 5 µm (right). (**B**) Myc-tagged *cnrN¯/CnrN^OE^* cells were incubated with rAprA, rCfaD, both rAprA and rCfaD, or an equivalent volume of buffer and lysed at the indicated times. Membranes from the lysates were collected by centrifugation and were analyzed for the presence of myc-tagged CnrN by Western blotting and staining for the myc-tag. Band intensities were measured as a ratio of the zero time point. Values are mean ± SEM, n = 3 or more. * and *** indicate a significant difference with p<0.05 or p<0.001, respectively, compared to the zero time point (t-test). (**C**) Membrane samples were run on a 4-20% PAGE gel and coomassie stained as a loading control.

PTEN can translocate from the cytosol to the inner face of the plasma membrane in response to extracellular signals [11]. We observed CnrN localization at the periphery of cells, in the interior of cells, and on vesicle-like structures in the interior of cells (Figure 4A). To test whether CnrN translocates in response to extracellular AprA or CfaD, we examined the localization of myc-tagged CnrN in the *cnrN¯* mutant background. Since both the ectopically expressed CnrN and our recombinant proteins are myc-tagged, we used immunoblotting of partially purified membranes to specifically examine CnrN levels. Log-phase *cnrN¯/CnrN^OE^* cells were stimulated with rAprA, rCfaD, a mix of rAprA and rCfaD, or buffer for 10 and 20 minutes. The cells were then lysed, and Western blots of the membranes were stained with antibodies against the myc tag. At ten minutes after stimulation, all conditions showed similar amounts of membrane-associated myc-CnrN compared to the zero time point (Figure 4B). After 20 minutes of stimulation with AprA alone or CfaD alone, there was no significant effect on the amount of membrane-associated myc-CnrN (Figure 4B). A significant increase in membrane-associated myc-CnrN was observed at 20 minutes when cells were stimulated with both AprA and CfaD (Figure 4B). For unknown reasons, the amount of membrane-associated myc-CnrN decreased at 20 minutes in the buffer control (Figure 4B). These results suggest that AprA and CfaD together can induce localization of CnrN to the membrane.

### CnrN is necessary for chemorepulsion by AprA

**Figure 5:**
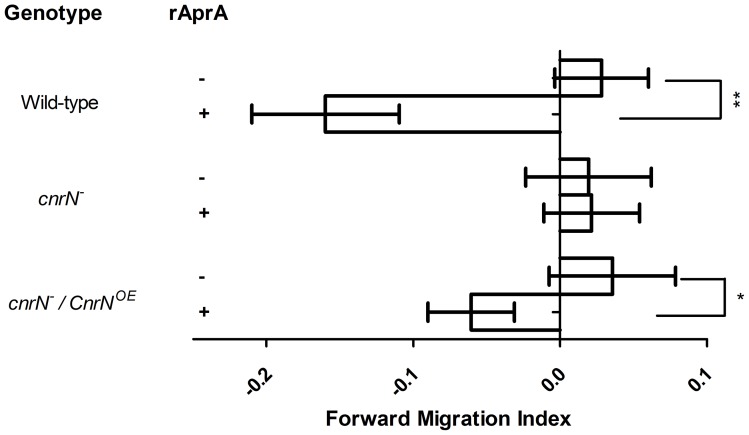
CnrN is necessary for chemorepulsion by AprA. Cells were assayed for chemorepulsion in response to rAprA in an Insall chamber for 1 hour. A negative migration index indicates movement away from the AprA source. Values are mean ± SEM of at least three independent experiments. * (p<0.05) and ** (p<0.01) indicate a significant difference (t-test).

We previously characterized AprA as a chemorepellent of *Dictyostelium* cells [10]. To determine if CnrN is necessary for chemorepulsion by AprA, we used Insall chambers [10], [38] to analyze the response of *cnrN¯* cells to rAprA. The forward migration index represents the displacement of cells along a gradient as a fraction of the total cell movement. A negative forward migration index indicates displacement away from rAprA. The chemorepulsion of wild type cells away from rAprA was similar to previous observations [10]. *cnrN¯* cells were not chemorepulsed in a gradient of AprA, and expressing CnrN in the mutant background rescued this defect (Figure 5). This indicates that CnrN is required for AprA to act as a chemorepellent of *Dictyostelium* cells.

## Discussion

Extracellular AprA and CfaD inhibit *Dictyostelium* cell proliferation and extracellular AprA causes cell chemorepulsion [6], [9], [10]. *cnrN¯* cells are insensitive to both the proliferation-inhibiting and chemorepellent effects of rAprA, and these phenotypes are rescued by expressing CnrN in *cnrN¯* cells. Like *aprA¯* and *cfaD¯* cells, *cnrN¯* cells have a faster doubling time and reach a higher cell density than wild type cells, have similar mass and protein accumulation per nucleus, and form less viable spores. Like *aprA¯* and *cfaD¯* cells, *cnrN¯* cells are also more multinucleate than wild type cells. Interestingly, adding rCfaD to cells lacking CnrN increases proliferation. The increase in proliferation of *cnrN¯* cells in response to rCfaD appears to be due to an increase in cytokinesis. The significant difference in the increase of *cnrN¯* cell proliferation between the combination of rAprA and rCfaD, and rCfaD alone, suggests a cooperative role between AprA and CfaD. If AprA and CfaD function independently, *cnrN¯* cells would respond to the combination of rAprA and rCfaD similarly to rCfaD alone, since *cnrN¯* cells are insensitive to rAprA signaling. Thus, the presence of AprA decreases the ability of CfaD to increase the proliferation of *cnrN¯* cells. Together, the data suggest that CnrN is required for AprA and CfaD to inhibit proliferation.

PTEN-like phosphatases are recruited to the plasma membrane to dephosphorylate PIP_3_ to PIP_2_ [18], [30], [31], [32], [33]. CnrN is localized in the cytosol, in vesicle-like structures, and at the plasma membrane. PTEN binds to phosphoserine/phosphocholine lipid vesicles via the C2 domain of the protein [39]. Since CnrN contains a putative PTEN-like C2 domain [20], CnrN may also bind to vesicles containing phosphoserine and phosphocholine. We found that CnrN localization is increased on membranes in response to a mixture of rAprA and rCfaD after 20 minutes. AprA and CfaD may recruit CnrN to the membrane to inhibit proliferation-promoting factors. In addition, AprA may also utilize CnrN membrane recruitment to potentiate chemorepulsion of *Dictyostelium* cells. CnrN is a necessary component of the AprA chemorepellent pathway. During chemotaxis, PTEN inhibits the formation of pseudopods at the trailing end of a cell [13], [14], [15]. In the AprA chemorepellent system, the PTEN-like phosphatase CnrN may be recruited to the membrane adjacent to the source of AprA. This would then inhibit the formation of pseudopods in the direction of the AprA source, allowing cells to move away from the source of AprA.

Together, our results indicate that CnrN is recruited to the membrane in response to a combination of AprA and CfaD, acts as a negative regulator of proliferation, and mediates AprA-induced chemorepulsion. An intriguing possibility is that in higher eukaryotes, factors that slow proliferation and/or act as chemorepellents may also regulate the localization of PTEN or PTEN-like phosphatases.
